# The Influence of the Interaction between Climate and Competition on the Distributional Limits of European Shrews

**DOI:** 10.3390/ani12010057

**Published:** 2021-12-28

**Authors:** Tomé Neves, Luís Borda-de-Água, Maria da Luz Mathias, Joaquim T. Tapisso

**Affiliations:** 1 CESAM—Centro de Estudos do Ambiente e do Mar, Departamento de Biologia Animal, Faculdade de Ciências, Universidade de Lisboa, 1749-016 Lisboa, Portugal; jstapisso@fc.ul.pt; 2 CIBIO/InBio, Centro de Investigação em Biodiversidade e Recursos Genéticos, Laboratório Associado, Campus Agrário de Vairão, Universidade do Porto, 4485-661 Vairão, Portugal; lbagua@cibio.up.pt; 3 CIBIO/InBio, Centro de Investigação em Biodiversidade e Recursos Genéticos, Laboratório Associado, Instituto Superior de Agronomia, Universidade de Lisboa, Tapada da Ajuda, 1349-017 Lisbon, Portugal; 4 BIOPOLIS Program in Genomics, Biodiversity and Land Planning, CIBIO, Campus de Vairão, 4485-661 Vairão, Portugal

**Keywords:** biotic interactions, competition, environmental niche models, Joint Species Distribution Models, shrews, Soricidae, species distributions

## Abstract

**Simple Summary:**

It is known that species’ distributions are influenced by several ecological factors. Nonetheless, the geographical scale upon which the influence of these factors is perceived is largely undefined. We assessed the importance of competition in regulating the distributional limits of species at large geographical scales. We studied European Soricidae shrews, because their species have similar diets, and focused on how interspecific competition changes along climatic gradients. We used presence data for the seven most widespread terrestrial species of Soricidae in Europe, gathered from online repositories, European museums, and gridded climate data. Using two different methods, we analysed the correlations between species’ presences, aiming to understand the distinct roles of climate and competition in shaping species’ distributions. Our results support three key conclusions: (i) climate alone does not explain all species’ distributions at large scales; (ii) negative interactions, such as competition, seem to play a strong role in defining species’ range limits, even at large scales; and (iii) the impact of competition on a species’ distribution varies along a climatic gradient, becoming stronger at the climatic extremes. Our conclusions support previous research, highlighting the importance of considering biotic interactions when studying species’ distributions, regardless of geographical scale.

**Abstract:**

It is known that species’ distributions are influenced by several ecological factors. Nonetheless, the geographical scale upon which the influence of these factors is perceived is largely undefined. We assessed the importance of competition in regulating the distributional limits of species at large geographical scales. We focus on species with similar diets, the European Soricidae shrews, and how interspecific competition changes along climatic gradients. We used presence data for the seven most widespread terrestrial species of Soricidae in Europe, gathered from GBIF, European museums, and climate data from WorldClim. We made use of two Joint Species Distribution Models to analyse the correlations between species’ presences, aiming to understand the distinct roles of climate and competition in shaping species’ distributions. Our results support three key conclusions: (i) climate alone does not explain all species’ distributions at large scales; (ii) negative interactions, such as competition, seem to play a strong role in defining species’ range limits, even at large scales; and (iii) the impact of competition on a species’ distribution varies along a climatic gradient, becoming stronger at the climatic extremes. Our conclusions support previous research, highlighting the importance of considering biotic interactions when studying species’ distributions, regardless of geographical scale.

## 1. Introduction

Climate change is an ongoing reality, making it a current priority in conservation biology to forecast how species might respond to climatic alterations [1,2]. To achieve this, it is imperative to first establish the factors that control the limits of species’ distributions at all geographic scales.

It is known that there is a variety of ecological factors that influence species’ geographical distributions, such as abiotic conditions and biotic interactions [3]. However, the latter has only recently been acknowledged as playing a key role in shaping species’ ranges [4,5], although the scale at which it does is still debatable. It has been previously suggested that biotic interactions only play a role at relatively local scales, but recent studies have argued that this is not always the case [6,7,8].

The uncertainty of the scale at which biotic interactions influence species’ distributions is problematic. At a local scale, negative interactions, such as competition, predation, or parasitism, can all lead to reduced abundance or even exclusion of a species from a given area [9]. Inversely, mutualistic interactions can allow species to extend their range into areas that would otherwise be inhospitable [10]. Disregarding any of these situations when forecasting species distributions will likely cause a large mismatch between the realized species’ niche and their predicted niche. Worse still, the strength and direction of biotic interactions are not insensitive to environmental change, as they have been shown to vary both over temporal and spatial environmental gradients, further complicating predictions under climate change [11,12,13,14].

Most of the large-scale studies focusing on the impact of biotic interactions involve plants [15,16,17], insects [18,19], or species associated with different trophic levels [20,21]. The work by Leach et al. (2017) is one of the few studies that, following a modelling approach, analysed competition between closely related mammalian species (lagomorphs) at a continental scale, although models accounting for the interaction between environment and biotic interactions were not considered [22]. To fill this void, here we explore the relative impact of competition between closely related species and climate in defining species’ distributional limits at a continental geographical scale. To this effect, we make use of two different complementary Joint Species Distribution Models (JSDMs) [18,23], to examine, as a case study, the distribution of European shrews. One of these models evaluates whether environmental variables alone justify species co-occurrence patterns [23], while the other assesses how the correlation between species’ pairs changes across environmental gradients [18].

**Figure 1:**
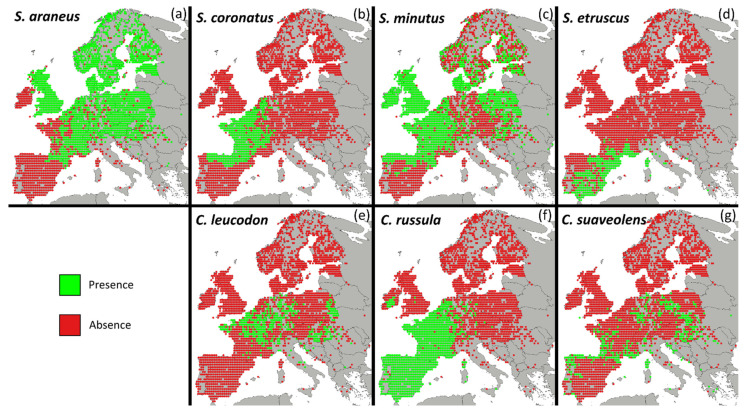
European distribution of the seven Soricidae species: (**a**) *Sorex araneus*; (**b**) *S. coronatus*; (**c**) *S. minutus*; (**d**) *Suncus etruscus*; (**e**) *Crocidura leucodon*; (**f**) *C. russula*; and (**g**) *C. suaveolens*. Green represents presence and red absence. The grey background map is solely for visual reference. Presences are organized on a grid of 35 km × 35 km where only cells where at least one species was present were considered. Thus, all coloured (green and red) cells are green for at least one species. The map projection is World Mollweide.

We have previously shown that competition among members of two subfamilies of Soricidae (order Eulipotyphla), the Crocidurinae and Soricinae, plays a role in controlling their global distribution [24]. There are about 360 species belonging to the two subfamilies spread across most of the globe, occurring from local to continental scales in allopatry, parapatry or sympatry [25]. These two subfamilies are well-represented in Europe, where the seven most widespread terrestrial species are *Crocidura leucodon*, *Crocidura suaveolens*, *Crocidura russula*, *Sorex araneus*, *Sorex coronatus*, *Sorex minutus,* and *Suncus etruscus* [26]. The distribution of these seven species varies in both total range and location. *S. minutus* and *S. araneus* are the most widespread across Europe, absent solely in most of the Iberian Peninsula. Other than these two, none of the mentioned species occur in northern Europe. However, all seven species occur in Western Europe, albeit occupying different ranges. *C. russula* occurs everywhere west of Germany, but the other four species have patchier distributions. This means that all the seven species have distributions that in some way overlap in Europe [26]. Focusing on the limits of the species’ distributions (Figure 1), we can distinguish what appear to be different physical and ecological barriers to their distributional limits. Some distributional limits have clear physical barriers as their origins, such as the limits in northern Italy, due to the Alps, or those of the British Isles, due to the North Sea. Here, however, we focus on species’ distributional limits with no obvious geographical cause, as those are the ones that can possibly be influenced by climate or competition. For example, *S. araneus*’ and *S. coronatus*’ distributions appear to complement one another, as their distributions barely intersect, while their limits nearly overlap, a pattern indicative of competitive exclusion. Other limits, however, show no clear transitions of one species into another. Such is the case for *S. coronatus* and *S. minutus*, whose distributional ranges in the Iberian Peninsula end at approximately the same latitude with no obvious geographical barrier, a pattern indicative of a climatic barrier. In addition to these two extremes examples, several of the distributional limits of the seven species fall into situations somewhere in between.

Taking into account the distributional overlap of the seven species, the existing evidence of competition, and that they share a similar diet [27,28], we expect some of the continental distributional limits of these species to be defined by competition, climate, and a combination of the two.

In order to support this possibility, we employ a combination of two different models along with the study of the existing literature on the species’ biology. Our analyses are performed at the European scale and with a relatively coarse resolution so that we can focus on the absolute range of the species, not on possible gaps in their local distribution.

Thus, our approach aims to establish if, at a continental scale, the various aforementioned barriers to the species’ distribution limits have climatic or biotic roots (or even, possibly, a combination of the two). If competition between closely related species is a driving factor of the apparent large-scale barriers to species’ distribution boundaries, we expect to find negative correlations between species’ pairs that cannot be fully explained by climate. As such, we expect to exemplify the importance of considering competition and climate simultaneously when forecasting species’ response to climatic alterations, regardless of the geographical scale, and, in particular, to improve the knowledge on the factors that control the distribution of Soricidae shrews in Europe.

## 2. Materials and Methods

### 2.1. Species Data

We collected presence data for the seven most widespread terrestrial species of Soricinae in Europe (*Crocidura leucodon*, *Crocidura russula*, *Crocidura suaveolens*, *Sorex araneus*, *Sorex coronatus*, *Sorex minutus* and *Suncus etruscus*) from the Global Biodiversity Information Facility [29], European museums, and other sources (Anděra, 2011 and Supplementary Materials Table S1) (Figure 1) [30]. The two largest species, by average body weight, are *C. russula* (12 g) and *C. leucodon* (11.7 g). Inversely, the two smallest are *S. etruscus* (2.3 g) and *S. minutus* (4.4 g). The other three species sit in between and are similar in size, with *S. coronatus* weighing 9.1 g, *S. araneus* 8.1 g, and *C. suaveolens* 7.6 g [31]. Although the size of the species’ prey is correlated with their body size, the seven species share a similar niche with regard to prey selection [27,28].

Almost all of the records we obtained from museums only mentioned the locality where the specimens were collected and had to be georeferenced manually. To do this, we obtained the coordinates of the centre of the locality using Google Maps Geocoding API [32]. Since our objective was to solely analyse the limits of the species’ distributions, the presences were forced into a square grid over Europe with each cell measuring 35 km by 35 km. Given this coarse resolution, the correlations obtained will be solely based on the range of the species’ distributions and only inform about how the presence of other species influences the limits of a given species. We used the centroid of each cell for the analysis. Only cells containing at least one presence from one species were considered, to help avoid false absence errors due to lack of sampling. This strategy incorporates the survey effort into the selection of the pseudo-absences required by the model by making sure that only cells that were sampled are counted as absences [33]. To define the grid size, we used presences that had both a georeferenced point and a locality and we measured the distance between the presences’ georeferenced points and the locality centre obtained through the Google Maps Geocoding API. We then chose a grid size that was bigger than the obtained distances to ensure that the coordinates we estimated for presences with only the locality data likely fell on the same grid as the real presences. In this way, the resolution used serves to reduce the problem of poorly georeferenced presence data.

### 2.2. Environmental Variables

**Figure 2:**
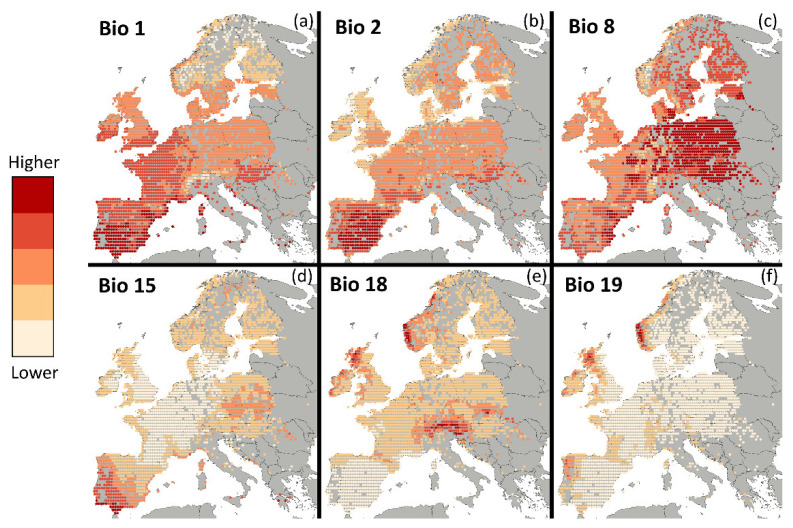
Values of the 6 climatic variables: (**a**) Annual Mean Temperature (Bio 1), (**b**) Mean Diurnal Range (mean of the monthly maximum temperature subtracted by the monthly minimum temperature) (Bio 2), (**c**) Mean Temperature of Wettest Quarter (Bio 8), (**d**) Precipitation Seasonality (Bio 15), (**e**) Precipitation of Warmest Quarter (Bio 18), and (**f**) Precipitation of Coldest Quarter (Bio 19). Darker tones represent higher values of the variable. The values are climatic normals from 1960 to 1990, obtained from WorldClim at a resolution of 10 arcminute and downsampled to the same resolution as the presences (35 km × 35 km) by averaging the values inside each grid. The colour scale divides the total range of each variable in five sections of equal range. The map projection is World Mollweide.

We obtained gridded climate data on 19 variables (Supplementary Materials Table S2), averaged from 1960 to 1990, from WorldClim [34] at a resolution of 10 arcminute. This is the largest spatial grain available from WorldClim, chosen to better approximate the resolution of the grid used to establish presences. Even so, we downsampled the data to the same resolution as the grid of presences by averaging the values inside each grid. To prevent multicollinearity, we computed a regression analysis and removed variables one by one, starting with the one with the highest Variation Inflation Factor (VIF), until the VIF of all the variables used was inferior to 5 [35]. The six remaining variables were Annual Mean Temperature (Bio 1), Mean Diurnal Range (mean of the monthly maximum temperature subtracted by the monthly minimum temperature) (Bio 2), Mean Temperature of Wettest Quarter (Bio 8), Precipitation Seasonality (Bio 15), Precipitation of Warmest Quarter (Bio 18), and Precipitation of Coldest Quarter (Bio 19) (Figure 2).

### 2.3. Modelling Approach

Here we use two different complementary JSDMs, one by Pollock et al. (2014) [23] and the other by Clark et al. (2018) [18], to understand the relative importance of climate and competition in shaping the distribution of European shrews.

Pollock’s framework produces two outputs. One depicts the correlation between pairs of species that can be attributed to a similar climatic niche. The other shows the residual correlation between pairs of species, i.e., the correlation that cannot be explained by the climatic variables. A strong residual correlation suggests that some type of biological interaction takes place between the species, albeit it may also be due to the model lacking the correct explanatory variable.

Clark’s framework reveals how the correlation between each pair of species changes across climatic gradients. The correlation between species is presented as symmetrical regression coefficients that provide the impact of the presence/absence of a species on the log-odds of the occurrence probability of the other species. This means that if the log-odds of the occurrence probability of species A is modified by the presence/absence of species B by α, then the presence/absence of A also modifies the log-odds of the occurrence probability of species B by α. Positive regression coefficients indicate that the species pair co-occurs more than would be expected by chance, while negative regression coefficients indicate that the species pair co-occurs less than expected. Additionally, this model also provides a measure of importance for the climatic variables and the presence/absence of other species when predicting the distribution of each species. To account for non-linear responses of the species to the temperature-related environmental variables (Supplementary Materials Figure S1), we included in this model the quadratic form of these three variables.

We ran both models in R 3.4.3 [36] using the codes provided in Clark et al. (2018) [18] and Pollock et al. (2014) [23]. Our code, along with the relevant parameters for both models, can be found in Data S1.

## 3. Results

Performing Pollock’s analysis (Table 1) reveals that, except for *Suncus etruscus* and *Sorex minutus*, all species pairings are significantly climatically correlated at a 95% confidence level, thus implying that climatic filtering is an important factor for justifying the co-occurrence (or segregation) of each species’ pair. As all correlations are positive, with the exception of the pairings *Sorex araneus* with *S. etruscus*, *Sorex coronatus*, *Crocidura suaveolens*, and *Crocidura russula*, most of the species’ pairs share similar climatic niches. However, not all species’ pairs have a significant residual correlation. Only *S. araneus* has a significant negative residual correlation with other species (*S. coronatus*, *C. russula*, and *Crocidura leucodon*). Additionally, several other pairings have significant positive residual correlations between them, indicating that more factors other than the climatic variables are influencing the co-occurrence patterns of the species pairs.

**Figure 3:**
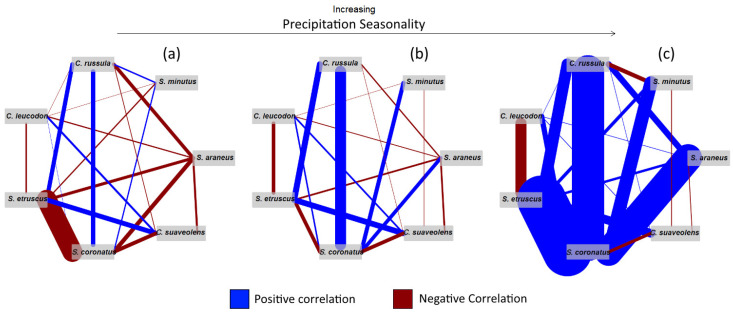
Results using Clark’s model. Correlation between pairs of species at the (**a**) 5th percentile, (**b**) 50th percentile, and (**c**) 95th percentile value of Precipitation Seasonality (Bio15). All other variables are at their average. The width of the lines represents correlation strength. Blue lines represent positive correlations, and red lines represent negative correlations.

Running Clark’s analysis led to several tables (Figure 3, Supplementary Materials Tables S3–S11, Supplementary Materials Figures S2–S6), detailing how the correlation between pairs of species varied along the climatic gradient. These results clearly show how changes in the climate strongly influence the co-occurrence patterns between species pairs, as revealed in the drastic difference found between the right and left networks of Figure 3 (and of Supplementary Materials Figures S2–S6). For example, *S. etruscus* and *S. coronatus* presences have an impact on the log-odds of the presence of the other by 8.174 at the 95th percentile of Precipitation Seasonality and by −8.457 at the 5th percentile, a difference of 16.63. Not all co-occurrence patterns between species pairs vary as wildly with changes in this climatic variable, though. In the case of *S. minutus* and *C. leucodon*, for instance, the fluctuation of the log-odds is a negligible 0.025 (−0.120 at the 5th percentile and −0.096 at the 95th) for the same variable (Supplementary Materials Table S9).

Clark’s model led to seven more tables, one per species, detailing the relative importance of each variable in predicting that species’ distribution (Supplementary Materials Tables S12–S18). Here, we provide a summary of these results. The distribution of some species is directly predicted by climatic variables. This is the case for *S. coronatus*, which favours lower values of Precipitation Seasonality, and for *S. araneus* and *C. suaveolens*, which prefer lower and higher values of Annual Mean Temperature, respectively. The presence of another species can also be a constant predictor of the presence (or absence) of a species, independently of the climatic variables. For example, *S. coronatus* and *S. etruscus* both have their distributions positively predicted by the presence of *C. russula*, with the reverse also being true. In addition, *S. minutus* and *C. suaveolens* are positively predicted by the presence of *S. coronatus* and *S. etruscus*, respectively. However, the presence of a species does not necessarily constantly predict the presence (or absence) of another one. In fact, the distribution of some species can be either positively or negatively predicted by the presence of one other species, with the direction of this interaction changing along the gradient of a climatic variable. This was the case for all species. In the case of *S. araneus*, for example, the presence of *S. coronatus* can predict its presence in high values (95th percentile) of Precipitation of Warmest Quarter or predict its absence in low values (5th percentile). It is worth highlighting that all species had another species’ presence/absence as their best, or at least second-best, predictor (Tables S12–S18).

## 4. Discussion

Our analyses of seven species of Soricidae shrews revealed that while climate is obviously important in determining the limits of a species’ distributions at large biogeographical scales, biotic interactions, particularly competition, also seem to play an important role. Additionally, we revealed that the importance of competition varies along the climatic gradient, being particularly relevant at climatic extremes.

Our study offers a sound basis on which to review and interpret the existing literature on the biology of these seven species, which, in combination, allow for a strong assessment of the relative importance of climate and competition to these species’ distributions. To guide our interpretation of the results, we took into consideration a recent study [37], which highlighted a pair of characteristics of common JSDMs. One is that the strength of the correlation for each species pair is significantly influenced by the prevalence of both species. Therefore, when comparing the correlation between different pairs of species with distinct prevalence, as in the present study, one should focus more on the significance of the correlations obtained than a direct comparison of their values. The other characteristic is that as the spatial resolution used in the JSDM grows coarser, the correlation strength between each species pair grows weaker, and that this is especially true for negative correlations, which disappear considerably faster than positive ones. As Clark’s model provides a single value for the correlation between a species pair, a decrease in the strength of the negative correlations, associated with the coarser resolution, forces the output of the model to be biased towards positive values. Considering this, we attribute a higher importance to negative correlations than to positive ones, since only strong significant negative correlations should persist at the coarse resolution used.

### 4.1. The Role of Climate

In the present study, the European distribution of *Sorex araneus* illustrates the importance of climate through its negative climatic correlation with most of the other species in Pollock’s model (Table 1). This result was to be expected, since *S. araneus* favours cold climates with distributions expanding to the north of Europe. The other species that reaches as far north is *Sorex minutus*, with which *S. araneus* shares a positive correlation (Figure 1, Table 1).

Environmental niche segregation related to climate is particularly evident between *S. araneus* and the two most southern species, *Crocidura russula* and *Suncus etruscus* (Table 1, Tables S12, S14 and S16). In fact, the two latter species are known to prefer warmer climates and are the only two in the present study that also occur in North Africa [38]. Such clear-cut climatic segregation is consistent with the evolutionary history of these three species and the energetic strategies they adopt to deal with different climatic conditions. *S. araneus* is hypothesized to have a Palearctic origin [39,40], where cold conditions favour the selection for high metabolic rates to maintain a constant body temperature and energy-saving mechanisms such as the Dehnel phenomenon [31,41]. On the contrary, both *C. russula* and *S. etruscus* are hypothesized to have originated in North Africa [40,42] where warmer environments favour selection for lower metabolic rates and the use of energy-saving mechanisms, such as daily torpor [31,43,44].

The pattern observed in France, of a turn-over from one species to another closely related, is relatively frequent in this area [45], even for different families, such as, for example, in the case of the voles *Arvicola amphibius* and *Arvicola sapidus*. Similar to our current case study, there is a species that occurs in the Iberian Peninsula and parts of France (*A. sapidus*) and another species that occurs throughout the rest of Europe (*A. amphibius*). Both *A. sapidus* and *C. russula* have originated in the Iberian Peninsula or Northern Africa, while *A. amphibious* and *S. araneus* have evolved in the northern parts of Europe and are thus better acclimated to the cold climates present there [42,45,46,47]. It is then likely that this pattern of segregation, with the corresponding apparent barrier somewhere around France, is the product of the evolutionary history of these species and climate.

Taking into account the projected increase in temperature in the coming years expected under climate change [1], one can expect this type of distributional barrier to shift towards northern Europe. As such, while the southern species will likely increase their range in the future, the northern ones’ range will probably decrease, at least if one ignores other possible ecological factors.

### 4.2. The Role of Competition

In addition to climate, biotic interactions can also play a role in defining species’ distributions. In fact, other species’ presence/absence were amongst the most important explanatory variables for all seven species’ distributions. We detected three pairs of negative residual correlations, implying the existence of competition, all involving *S. araneus* (*S. araneus* versus *Sorex coronatus*, *C. russula*, and *Crocidura leucodon*) (Table 1). Because two of these species, *S. coronatus* and *C. russula*, are present in the south-western limit of *S. araneus*’ distribution range (Figure 1), we suggest that the apparent barrier to *S. araneus*’ distribution in this region is a product of competition, in addition to climate. For one of these pairs, *S. araneus* and *S. coronatus*, competitive parapatry was already locally confirmed by Neet and Hausser (1990) [48], who reported the coexistence of both species, although exhibiting microhabitat segregation.

*C. leucodon* also showed a weak, albeit nearly constant, negative correlation with *S. araneus* (Table 1 and Supplementary Materials Tables S3–S11). This suggests that, along with *S. coronatus* and *C. russula*, *C. leucodon* might be limiting the southern distribution of *S. araneus*. Interestingly, considering the distinctive absence of *C. leucodon* near Poland, it also seems possible that *S. araneus* is limiting *C. leucodon*‘s northern distribution. Actually, *C. leucodon* is known to vary habitat preferences along its distributional range [26], but always occurring in habitats that *S. araneus* usually avoids. These results, highlighting the direct impact of competition in limiting species’ distributions, regardless of climate, fall in line with several other previous studies, showing how biotic interactions directly impact species’ distributions [22,49,50,51].

There are known cases of competition between pairs of species that were not detected in our models, sch as *S. araneus* with *S. minutus* [52,53,54,55], *C. russula* with *S. coronatus* [56], and *S. minutus* with *S. coronatus* [57]. In these cases, the distributions of the species almost completely overlap at the resolution of our analysis (Figure 1). Thus, even if microhabitat segregation occurs between these species, this does not appear to limit their distribution at a continental scale. One possibility is that competition between these species leads to a temporal segregation, instead of a geographical one. In fact, there already exists evidence of temporal segregation between members of this family, with species occupying the same location while being active at different times [58].

We also found positive correlations, as exemplified by the distribution of *C. russula*, which is strongly predicted by the presence of both *S. coronatus* and *S. etruscus* (Supplementary Table S14). In fact, we expected positive residual correlations for two reasons. First, positive correlations are easier to detect at the coarse resolution we employed, as the signal for negative correlations is often lost at these resolutions [37]. Second, and more important, while some of these species favour different climates, they all have a clear preference for the same type of habitat and conditions. For example, high soil humidity and prey availability are two factors that are extremely important for high within-habitat diversity and shrew abundance, regardless of the species [39]. As both variables cannot be accurately measured at the resolution employed here and thus were not included in our model, there is little doubt that the presence of a species is serving as a proxy variable for suitable habitats for the other species. This is especially so given that the species we modelled share a similar niche regarding prey selection, with comparisons between the smaller and largest species revealing as much as 80% overlap of their diet [27,28]. Therefore, along with the inexistence of any evidence of mutualism between members of this family, we refrain from linking the obtained positive residual correlations to any kind of biotic interactions. Conversely, though, it is this same dietary overlap, along with shelter availability, that fuels the competition for resources responsible for the negative correlations we detected [59,60,61].

### 4.3. Changes in Biotic Interactions along Climatic Gradients

The importance of competition and the variation of its relevance along the climatic gradient in defining the limits of species’ distributions, with competition usually gaining importance at the climatic extremes, is illustrated by *S. etruscus*’, *C. leucodon*’s, and *S. coronatus*’ distributions. For example, *S. coronatus* appears to prefer constant precipitation throughout the year (Figure 1 and Figure 2, Supplementary Materials Table S17) and, once again, as seasonality decreases, its correlation with *S. etruscus* and *C. leucodon* decreases considerably (Supplementary Materials Table S9 and Figure 3). This is in line with what is known about *S. coronatus* and *S. etruscus*. The *Sorex* genus members prefer humid environments [26,48,62], while members of the *Suncus* genus favour drier environments [26,62,63]. *S. coronatus’* strong negative correlation with Precipitation Seasonality is then a consequence of the importance of constant precipitation throughout the year to maintain the humid environments where it thrives. It appears then that *S. etruscus* and *C. leucodon* do not co-occur as frequently with *S. coronatus* when climate is either favourable to *S. coronatus* or unfavourable to *S. etruscus* and *C. leucodon*. We suggest that these species could potentially expand into *S. coronatus*’ range but appear to be prevented from doing so due to unfavourable climatic conditions coupled with added competitive pressure.

The pair *S. minutus* and *C. russula* is also peculiar. Although we know they compete in Ireland [64] and roughly share the same diet [65,66], we found no major signal supporting competition between these species. For the entirety of the climatic gradient, the absolute value of the regression coefficient provided by Clark’s model never goes above 1. The strongest negative correlation detected was at the higher values of Precipitation Seasonality, where it reaches −0.77 (Supplementary Materials Table S9). This is likely due to the climate in the Iberian Peninsula and Southern France, where precipitation seasonality is high, *C. russula* is present, and *S. minutus* is largely absent. This happens despite *S. minutus’* relatively uniform distribution throughout the rest of Europe, where precipitation seasonality is also high, but *C. russula* is not present. This suggest that *S. minutus* might be able to expand to the entirety of the Iberian Peninsula and Southern France given the absence of *C. russula*, as it appears to be limited by the combination of both the presence of *C. russula* and unfavourable climatic conditions.

Interestingly, this is far from the only case where this apparent barrier to species’ distributions in the northern Iberian Peninsula manifests itself. In addition to shrews, there are several pairings of closely related species where one species is present throughout most of the Iberian Peninsula and the other only occurs in its northern part. This is, for example, the case with *Talpa europae* and *T. occidentalis*, *Lepus europaeus* and *L. granatensis*, and *Apodemus flavicollis* and *A. sylvaticus* [25]. Further research in this region would then seem warranted so as to ascertain which of these are sole products of climate and which are also influenced by competition.

Regardless, it seems clear that the relation between species is not constant throughout their distribution, and that climate might play a key role in defining how they interact. It is nonetheless possible that other factors allow the coexistence of the species, such as abundant food supply or different types of landscapes facilitating microhabitat segregation.

## 5. Conclusions

We aimed to ascertain the importance of biotic interactions in defining the continental distributional limits of species with similar diets, focusing on competition in particular, using as an example seven shrew species. Our main findings are: (i) climate alone does not explain the limits of all species’ distribution; (ii) there is evidence that competition, even among species with similar diets, can play an important role in defining a species’ distribution boundaries at large geographical scales; and (iii) the strength of the competition is a function of the climatic gradient, frequently becoming strongest at the climatic extremes, which do not necessarily correlate to the species’ geographic extremes of occurrence.

Our conclusions support earlier studies establishing the importance of considering biotic interactions when studying species’ distributions, especially when projecting to future climatic conditions [20,21,22,67]. Here, building on these conclusions, we demonstrate how different climatic conditions can change both the direction and strength of this type of competition. This makes it possible, and maybe even likely, that species’ distributions may change unpredictably under climate change, if the biotic interactions underlying the limits of the species’ distributions are not well-understood [68]. Therefore, our results stand as further warning that biotic interactions should be considered even between species with similar diets or at large geographical scales. Further, we highlight that these interactions should not be assumed to remain constant across climatic gradients, frequently gaining importance at climatic extremes, and thus require particular attention when dealing with climate change scenarios.

## Figures and Tables

**Table 1:** Results using Pollock’s framework. Results above the diagonal are for environmental correlation. Results below the diagonal are for residual correlation. Environmental correlation is the correlation between species that is explained by the climatic variables included in the model. Residual correlation is the correlation that is not explained by any variable included in the model.

Species	*S. etruscus*	*C.* *leucodon*	*C.* *russula*	*C.* *suaveolens*	*S.* *araneus*	*S.* *coronatus*	*S.* *minutus*
*S. etruscus*		0.312 *	0.906 *	0.775 *	−0.833 *	0.642 *	−0.099
*C. leucodon*	−0.109 *		0.310 *	0.495 *	0.209 *	0.543 *	0.622 *
*C. russula*	0.391 *	0.261*		0.722 *	−0.817 *	0.865 *	0.145 *
*C. suaveolens*	0.228 *	0.327 *	0.065		−0.541 *	0.613 *	0.209 *
*S. araneus*	0.038	−0.139 *	−0.111 *	−0.051		−0.490 *	0.278 *
*S. coronatus*	0.249 *	0.207 *	0.782 *	0.09	−0.251 *		0.602 *
*S. minutus*	0.018	0.138 *	0.296 *	0.173 *	−0.026	0.409 *	

* means significant at a 95% confidence level.

## Data Availability

The climate data is available from WorldClim at http://www.worldclim.org/, accessed on 17 May 2019. The code and presence data are in Data S1.

## Supplementary Materials

The following supporting information can be downloaded at: https://www.mdpi.com/article/10.3390/ani12010057/s1, Figure S1: Distribution of the seven species over the range of the several variables. Figure S2: Correlation between pairs of species at the 95th percentile (rightmost), 50th percentile (middle) and 5th percentile (leftmost) value of Annual Mean Temperature (Bio01). Figure S3: Correlation between pairs of species at the 95th percentile (rightmost), 50th percentile (middle) and 5th percentile (leftmost) value of Mean Diurnal Range (Bio02). Figure S4: Correlation between pairs of species at the 95th percentile (rightmost), 50th percentile (middle) and 5th percentile (leftmost) value of Mean Temperature of Wettest Quarter (Bio08). Figure S5: Correlation between pairs of species at the 95th percentile (rightmost), 50th percentile (middle) and 5th percentile (leftmost) value of Precipitation of Warmest Quarter (Bio18). Figure S6: Correlation between pairs of species at the 95th percentile (rightmost), 50th percentile (middle) and 5th percentile (leftmost) value of Precipitation of Coldest Quarter (Bio19). Table S1: Additional sources of presence data. Table S2: The 19 variables obtained from WorldClim. Table S3: Regression coefficients between pairs of species at the 95th percentile (Dark yellow) and 5th percentile (Light yellow) value of Annual Mean Temperature (Bio1). Table S4: Regression coefficients between pairs of species at the 50th percentile value of Annual Mean Temperature (Bio1). Table S5: Regression coefficients between pairs of species at the 95th percentile (Dark yellow) and 5th percentile (Light yellow) value of Mean Diurnal Range (Bio2). Table S6: Regression coefficients between pairs of species at the 50th percentile value of Mean Diurnal Range (Bio2). Table S7: Regression coefficients between pairs of species at the 95th percentile (Dark yellow) and 5th percentile (Light yellow) value of Mean Temperature of Wettest Quarter (Bio8). Table S8: Regression coefficients between pairs of species at the 50th percentile value of Mean Temperature of Wettest Quarter (Bio8). Table S9: Regression coefficients between pairs of species at the 95th percentile (Dark yellow) and 5th percentile (Light yellow) value of Precipitation Seasonality (Bio15). Table S10: Regression coefficients between pairs of species at the 95th percentile (Dark yellow) and 5th percentile (Light yellow) value of Precipitation of Warmest Quarter (Bio18). Table S11: Regression coefficients between pairs of species at the 95th percentile (Dark yellow) and 5th percentile (Light yellow) value of Precipitation of Coldest Quarter (Bio19). Table S12: Relative importance of variables in predicting the distribution of *Suncus etruscus*. Table S13: Relative importance of variables in predicting the distribution of *Crocidura leucodon*. Table S14: Relative importance of variables in predicting the distribution of *Crocidura russula*. Table S15: Relative importance of variables in predicting the distribution of *Crocidura suaveolens*. Table S16: Relative importance of variables in predicting the distribution of *Sorex araneus*. Table S17: Relative importance of variables in predicting the distribution of *Sorex coronatus*. Table S18: Relative importance of variables in predicting the distribution of *Sorex minutus*.
