# Supplementary material for: The Influence of the Interaction between Climate and Competition on the Distributional Limits of European Shrews
Source: Animals (Basel). 2021 Dec 28;12(1):57. doi: 10.3390/ani12010057 (PMC8749581; doi:10.3390/ani12010057)
Supplement: Supplementary file 1 [file animals-12-00057-s001.zip › Supporting Information S1.pdf]

SUPPORTING INFORMATION

The Influence of the Interaction between Climate and Competition on the Distributional Limits of European Shrews.

Tomé Neves[1,2,3,4*], Luís Borda-de-Água[2,3,4], Maria da Luz Mathias[1*], Joaquim T. Tapisso[1]

1 CESAM—Centro de Estudos do Ambiente e do Mar and Departamento de Biologia Animal, Faculdade de Ciências, Universidade de Lisboa, 1749-016 Lisboa, Portugal

2 CIBIO/InBio, Centro de Investigação em Biodiversidade e Recursos Genéticos, Laboratório Associado, Universidade do Porto, Campus Agrário de Vairão, 4485-661 Vairão, Portugal

3 CIBIO/InBio, Centro de Investigação em Biodiversidade e Recursos Genéticos, Laboratório Associado, Instituto Superior de Agronomia, Universidade de Lisboa, Tapada da Ajuda, 1349-017 Lisbon, Portugal

4 BIOPOLIS Program in Genomics, Biodiversity and Land Planning, CIBIO, Campus de Vairão, 4485-661 Vairão, Portugal

* Correspondence: tneves@fc.ul.pt (T.N.); mlmathias@fc.ul.pt (M.L.M.)

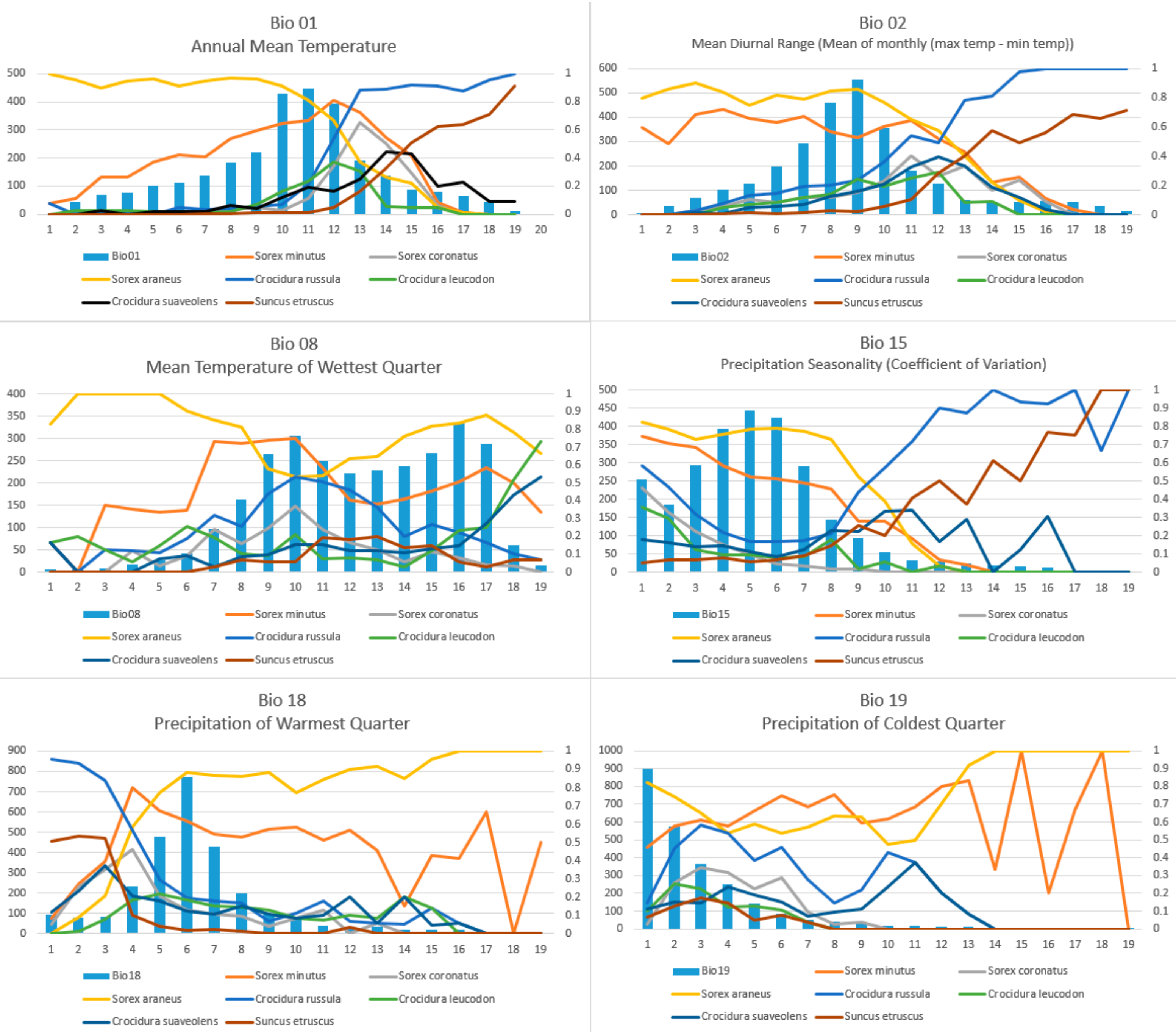

Supplementary Figure S1 – Distribution of the seven species over the range of the several variables. Each variable was divided into 19 classes of equal range, represented in the X axis, ordered by values. The blue bars (left Y axis) represent the number of cells of the presence map that fall under each of the 19 classes. The lines (right Y axis) represent the proportion of presences/absences for each species in the cells that fall under each of the 19 classes. The species are as follows: Sorex minutus *(orange);* Sorex coronatus *(grey);* Sorex araneus *(yellow);* Crocidura russula *(light blue);* Crocidura leucodon *(green);* Crocidura suaveolens *(dark blue); and* Suncus etruscus *(brown).*

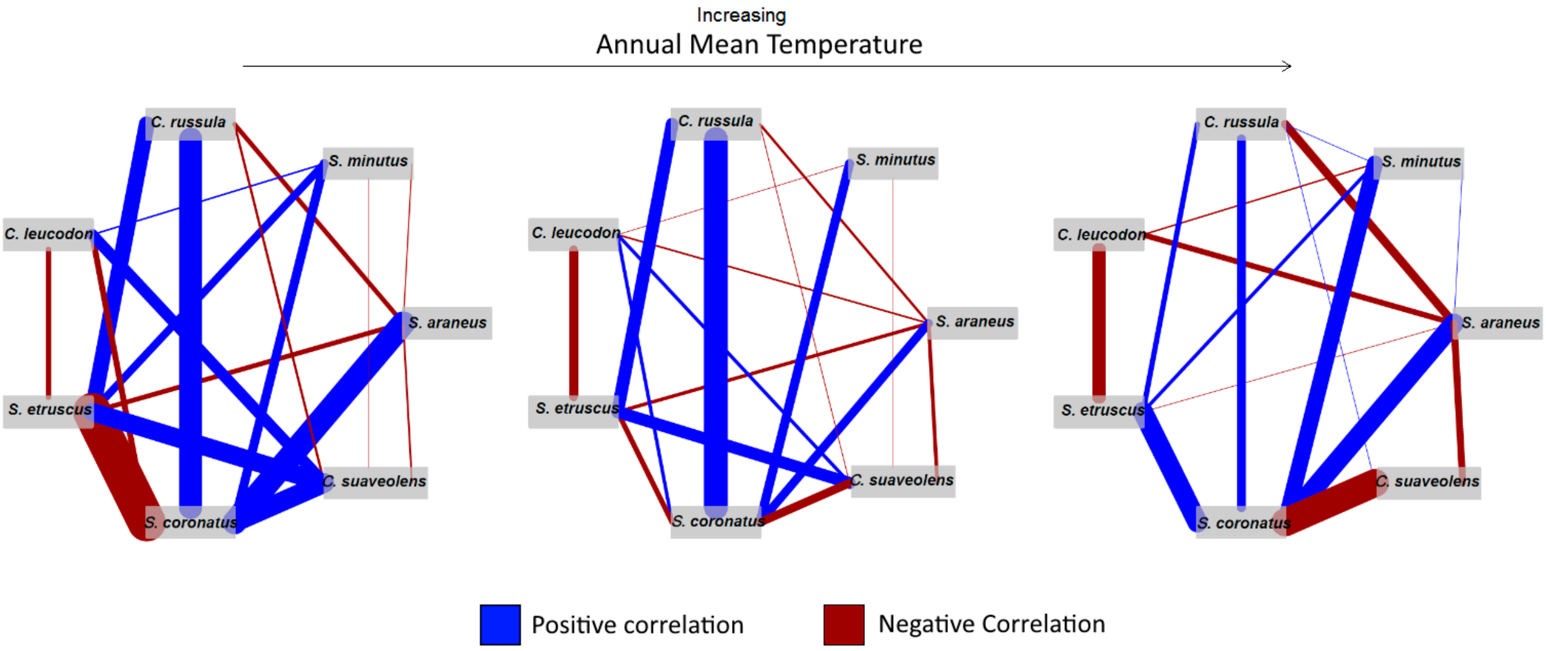

Supplementary Figure S2 - Correlation between pairs of species at the 95th percentile (rightmost), 50th percentile (middle) and 5th percentile (leftmost) value of Annual Mean Temperature (Bio01). All other variables are at their average. The width of the lines represents correlation strength. Blue lines represent positive correlations and red lines represent negative correlations.

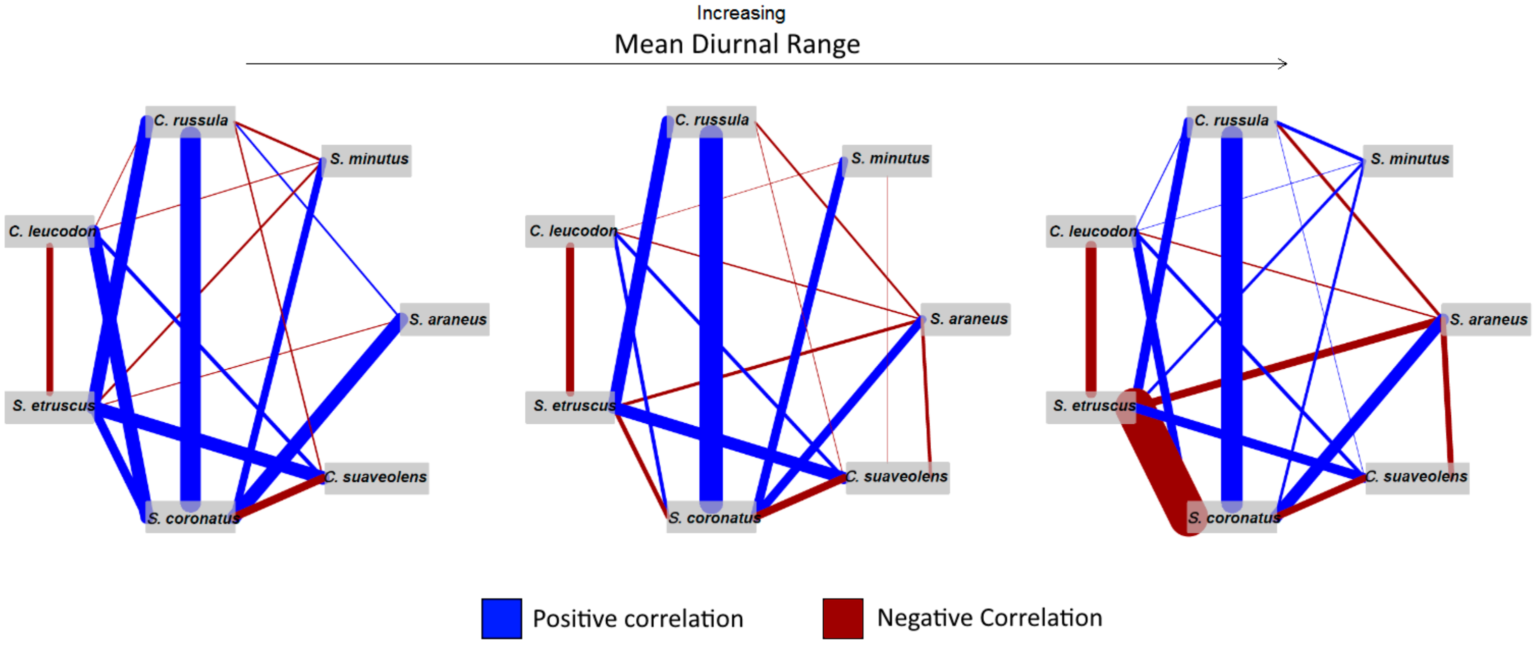

Supplementary Figure S3 - Correlation between pairs of species at the 95th percentile (rightmost), 50th percentile (middle) and 5th percentile (leftmost) value of Mean Diurnal Range (Bio02). All other variables are at their average. The width of the lines represents correlation strength. Blue lines represent positive correlations and red lines represent negative correlations.

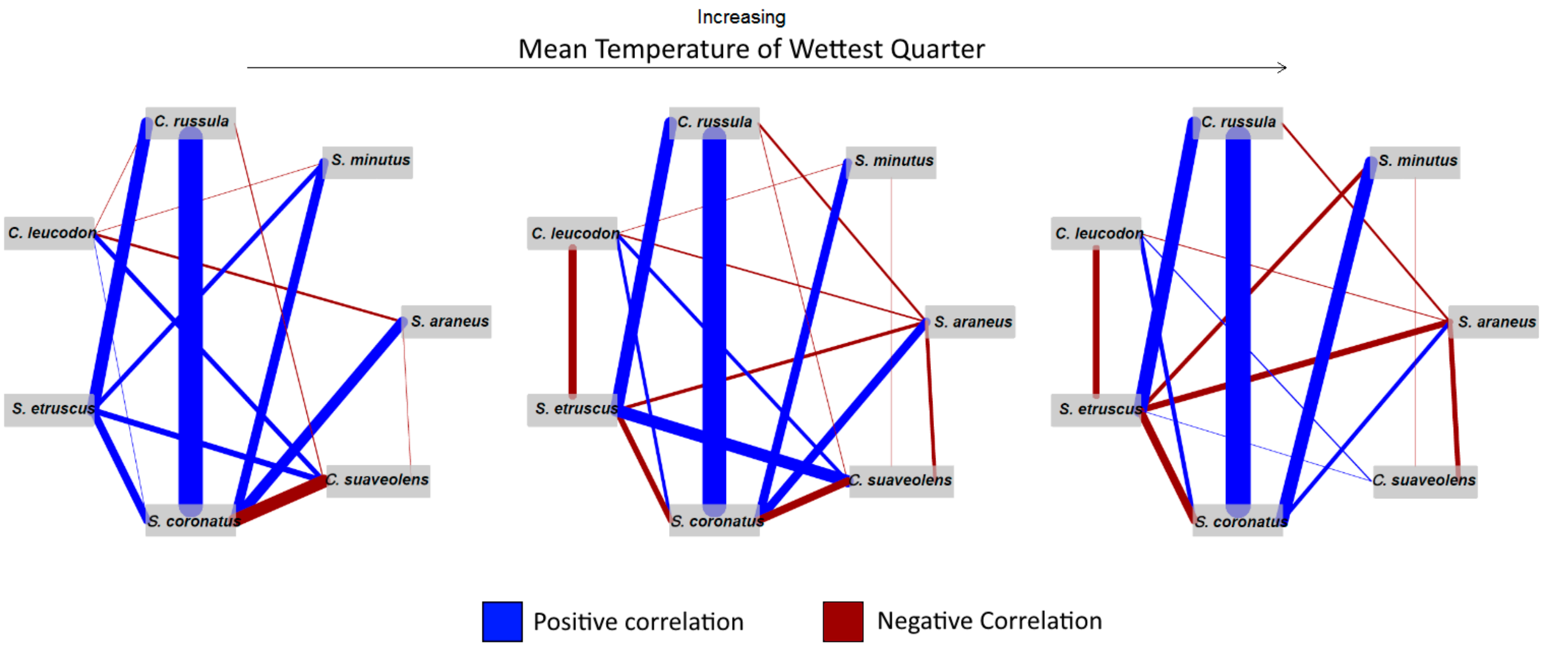

Supplementary Figure S4 - Correlation between pairs of species at the 95th percentile (rightmost), 50th percentile (middle) and 5th percentile (leftmost) value of Mean Temperature of Wettest Quarter (Bio08). All other variables are at their average. The width of the lines represents correlation strength. Blue lines represent positive correlations and red lines represent negative correlations.

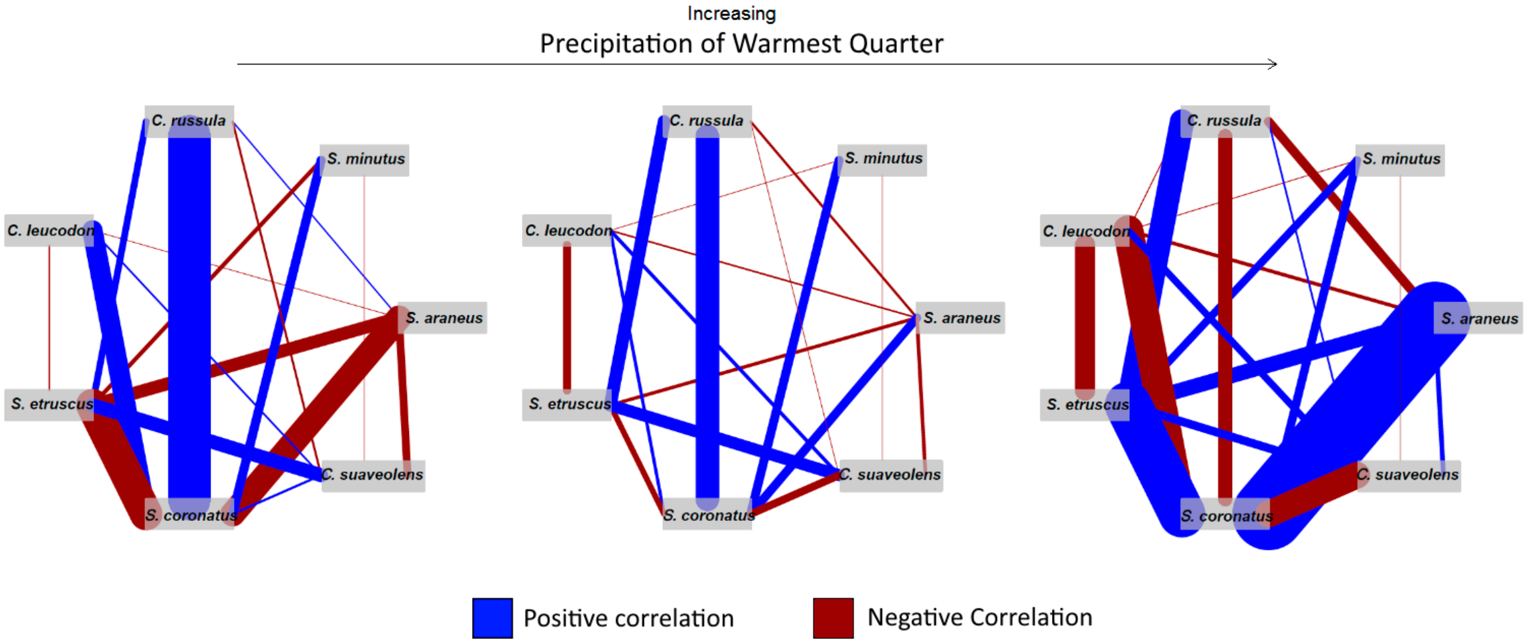

Supplementary Figure S5 - Correlation between pairs of species at the 95th percentile (rightmost), 50th percentile (middle) and 5th percentile (leftmost) value of Precipitation of Warmest Quarter (Bio18). All other variables are at their average. The width of the lines represents correlation strength. Blue lines represent positive correlations and red lines represent negative correlations.

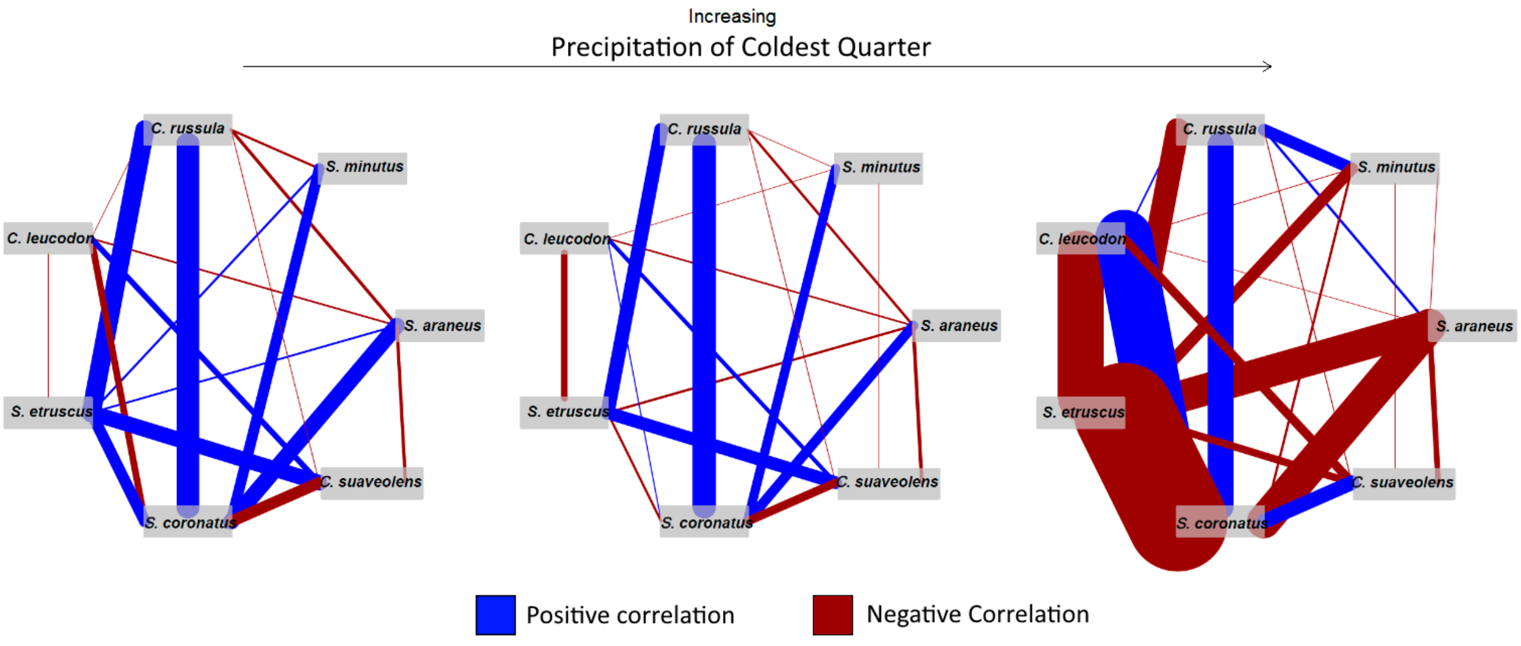

Supplementary Figure S6 - Correlation between pairs of species at the 95th percentile (rightmost), 50th percentile (middle) and 5th percentile (leftmost) value of Precipitation of Coldest Quarter (Bio19). All other variables are at their average. The width of the lines represents correlation strength. Blue lines represent positive correlations and red lines represent negative correlations.

Supplementary Table S1 – Additional sources of presence data.

Country	Institution	Link
Austria	Natural History Museum Vienna	https://www.nhm-wien.ac.at/
Belgium	Royal Belgian Institute of Natural Sciences	https://www.naturalsciences.be/
Czech Republic	National Museum, Prague	https://www.nm.cz/en/museum/science-and-research#news
Finland	Luomus - Finish Museum of Natural History	https://www.luomus.fi/en/zoological-collections
	Zoological Museum of the University of Oulu	https://www.oulu.fi/biodiversityunit/node/10713
	Kuopio Museum	https://kuopionluonnontieteellinenmuseo.fi/
France	French National Museum of Natural History	https://science.mnhn.fr/institution/mnhn/search
Germany	Natural History Museum, Berlin	https://www.museumfuernaturkunde.berlin/en/research/collections/mammals
Hungary	Hungarian Natural History Museum	http://www.nhmus.hu/en/collection/department_of_zoology/mammal_collection
Italy	Natural History Museum of Milan	https://web.comune.milano.it/dseserver/webcity/documenti.nsf/0/720f5d28caed28fac12571010056d065?OpenDocument&ExpandSection=2#_Section2
Netherlands	Naturalis Biodiversity Center	https://www.naturalis.nl/en/collection
Poland	Atlas Ssaków Polski	https://www.iop.krakow.pl/Ssaki/gatunki
Portugal	National Museum of Natural History and Science, Lisbon	https://museus.ulisboa.pt/pt-pt/colecao-mamiferos
Russia	State Darwin Museum	https://www.darwinmuseum.ru/
Serbia	Red List	http://www.nasljedje.org/prirodno-nasljedje/202

Spain	Atlas y Libro Rojo de mamíferos terrestres de España	https://www.miteco.gob.es/es/biodiversidad/temas/inventarios-nacionales/inventario-especies-terrestres/inventario-nacional-de-biodiversidad/ieet_mamif_atlas.aspx
	National Museum of Natural Sciences, Madrid	https://www.mncn.csic.es/es?idioma=es_ES&seccion=1311
Sweden	Swedish Museum of Natural History	https://www.nrm.se/en/forskningochsamlingar/zoologi/samlingar/vertebrater.9002804.html
Switzerland	Natural History Museum, Geneve	http://institutions.ville-geneve.ch/fr/mhn/notre-recherche/collections/des-collections-accessibles/collections-zoologiques-zootheque/chordata-mammalia-mammiferes/
United Kingdom	Natural History Museum, London	https://data.nhm.ac.uk/dataset/collection-specimens/resource/05ff2255-c38a-40c9-b657-4ccb55ab2feb

Supplementary Table S2- The 19 variables obtained from WorldClim.

**Variable**
Bio 1 - Annual Mean Temperature
Bio 2 - Mean Diurnal Range (Mean of monthly (max temp - min temp))
Bio 3 - Isothermality (BIO2/BIO7) (* 100)
Bio 4 - Temperature Seasonality (standard deviation *100)
Bio 5 - Max Temperature of Warmest Month
Bio 6 - Min Temperature of Coldest Month
Bio 7 - Temperature Annual Range (BIO5-BIO6)
Bio 8 - Mean Temperature of Wettest Quarter
Bio 9 - Mean Temperature of Driest Quarter
Bio 10 - Mean Temperature of Warmest Quarter
Bio 11 - Mean Temperature of Coldest Quarter
Bio 12 - Annual Precipitation
Bio 13 - Precipitation of Wettest Month
Bio 14 - Precipitation of Driest Month
Bio 15 - Precipitation Seasonality (Coefficient of Variation)
Bio 16 - Precipitation of Wettest Quarter
Bio 17 - Precipitation of Driest Quarter
Bio 18 - Precipitation of Warmest Quarter
Bio 19 - Precipitation of Coldest Quarter

Supplementary Table S3 - Regression coefficients between pairs of species at the 95th percentile (Dark yellow) and 5th percentile (Light yellow) value of Annual Mean Temperature (Bio1). All other variables are at their average. The regression coefficients are symmetrical and provide the impact of the presence/absence of a species on the log-odds of the occurrence probability of the other species.

	S. etruscus	*C. leucodon*	*C. russula*	*C. suaveolens*	*S. araneus*	*S. coronatus*	*S. minutus*
S. etruscus		-3.185	1.241	0.058	-0.142	4.823	0.807
C. leucodon	-1.196		-0.092	-0.058	-1.344	0.003	-0.335
C. russula	3.809	-0.058		0.122	-2.010	2.092	0.134
C. suaveolens	4.471	2.735	-0.540		-1.649	-6.754	-0.045
S. araneus	-1.127	0.096	-1.070	-0.421		4.695	0.137
S. coronatus	-9.229	-1.402	5.456	4.732	5.760		3.868
S. minutus	1.823	0.361	-0.040	-0.129	-0.251	2.161	

Supplementary Table S4 - Regression coefficients between pairs of species at the 50th percentile value of Annual Mean Temperature (Bio1). All other variables are at their average. The regression coefficients are symmetrical and provide the impact of the presence/absence of a species on the log-odds of the occurrence probability of the other species.

	S. etruscus	*C. leucodon*	*C. russula*	*C. suaveolens*	*S. araneus*	*S. coronatus*	*S. minutus*
S. etruscus		-1.874	3.057	2.882	-0.807	-1.203	-0.028
C. leucodon	-1.874		-0.075	0.787	-0.390	0.792	-0.119
C. russula	3.057	-0.075		-0.180	-0.563	5.614	-0.002
C. suaveolens	2.882	0.787	-0.180		-0.871	-2.014	-0.108
S. araneus	-0.807	-0.390	-0.563	-0.871		2.030	-0.004
S. coronatus	-1.203	0.792	5.614	-2.014	2.030		2.180
S. minutus	-0.028	-0.119	-0.002	-0.108	-0.004	2.180	

Supplementary Table S5 – Regression coefficients between pairs of species at the 95th percentile (Dark yellow) and 5th percentile (Light yellow) value of Mean Diurnal Range (Bio2). All other variables are at their average. The regression coefficients are symmetrical and provide the impact of the presence/absence of a species on the log-odds of the occurrence probability of the other species.

	S. etruscus	*C. leucodon*	*C. russula*	*C. suaveolens*	*S. araneus*	*S. coronatus*	*S. minutus*
S. etruscus		-2.471	2.346	2.316	-1.886	-9.542	0.662
C. leucodon	-1.540		0.253	0.869	-0.353	2.098	0.134
C. russula	3.264	-0.234		0.158	-0.754	5.189	0.830
C. suaveolens	3.252	0.913	-0.368		-1.421	-1.703	-0.018
S. araneus	-0.257	-0.033	0.426	0.095		2.940	0.065
S. coronatus	2.159	3.302	4.866	-1.938	3.347		0.674
S. minutus	-0.493	-0.278	-0.584	-0.064	-0.063	1.827	

Supplementary Table S6 – Regression coefficients between pairs of species at the 50th percentile value of Mean Diurnal Range (Bio2). All other variables are at their average. The regression coefficients are symmetrical and provide the impact of the presence/absence of a species on the log-odds of the occurrence probability of the other species.

	S. etruscus	*C. leucodon*	*C. russula*	*C. suaveolens*	*S. araneus*	*S. coronatus*	*S. minutus*
S. etruscus		-1.816	3.119	2.975	-0.770	-1.045	-0.058
C. leucodon	-1.816		-0.090	0.827	-0.353	0.877	-0.123
C. russula	3.119	-0.090		-0.205	-0.493	5.638	-0.052
C. suaveolens	2.975	0.827	-0.205		-0.794	-1.866	-0.109
S. araneus	-0.770	-0.353	-0.493	-0.794		2.079	-0.014
S. coronatus	-1.045	0.877	5.638	-1.866	2.079		2.168
S. minutus	-0.058	-0.123	-0.052	-0.109	-0.014	2.168	

Supplementary Table S7 – Regression coefficients between pairs of species at the 95th percentile (Dark yellow) and 5th percentile (Light yellow) value of Mean Temperature of Wettest Quarter (Bio8). All other variables are at their average. The regression coefficients are symmetrical and provide the impact of the presence/absence of a species on the log-odds of the occurrence probability of the other species.

	S. etruscus	*C. leucodon*	*C. russula*	*C. suaveolens*	*S. araneus*	*S. coronatus*	*S. minutus*
S. etruscus		-1.602	3.319	0.134	-1.537	-1.912	-0.989
C. leucodon	-0.086		-0.045	0.300	-0.218	1.017	-0.051
C. russula	2.985	-0.122		-0.064	-0.507	5.961	-0.004
C. suaveolens	1.432	1.109	-0.256		-1.187	-0.072	-0.166
S. araneus	-0.071	-0.598	-0.059	-0.137		0.974	-0.004
S. coronatus	1.879	0.125	5.692	-3.167	2.553		3.157
S. minutus	1.092	-0.143	-0.010	-0.056	0.028	2.285	

Supplementary Table S8 – Regression coefficients between pairs of species at the 50th percentile value of Mean Temperature of Wettest Quarter (Bio8). All other variables are at their average. The regression coefficients are symmetrical and provide the impact of the presence/absence of a species on the log-odds of the occurrence probability of the other species.

	S. etruscus	*C. leucodon*	*C. russula*	*C. suaveolens*	*S. araneus*	*S. coronatus*	*S. minutus*
S. etruscus		-1.835	3.093	2.956	-0.811	-1.370	-0.006
C. leucodon	-1.835		-0.075	0.829	-0.372	0.772	-0.110
C. russula	3.093	-0.075		-0.190	-0.543	5.667	-0.005
C. suaveolens	2.956	0.829	-0.190		-0.847	-1.879	-0.108
S. araneus	-0.811	-0.372	-0.543	-0.847		2.042	-0.009
S. coronatus	-1.370	0.772	5.667	-1.879	2.042		2.147
S. minutus	-0.006	-0.110	-0.005	-0.108	-0.009	2.147	

Supplementary Table S9 –. Regression coefficients between pairs of species at the 95th percentile (Dark yellow) and 5th percentile (Light yellow) value of Precipitation Seasonality (Bio15). All other variables are at their average. The regression coefficients are symmetrical and provide the impact of the presence/absence of a species on the log-odds of the occurrence probability of the other species.

	S. etruscus	*C. leucodon*	*C. russula*	*C. suaveolens*	*S. araneus*	*S. coronatus*	*S. minutus*
S. etruscus		-3.181	4.121	3.472	-0.086	8.174	0.590
C. leucodon	-0.858		0.020	0.634	-0.143	1.465	-0.096
C. russula	2.338	-0.144		-0.044	0.761	9.577	-0.763
C. suaveolens	2.558	0.964	-0.295		-0.674	-1.769	-0.202
S. araneus	-1.364	-0.536	-1.511	-0.986		6.487	0.005
S. coronatus	-8.456	0.271	2.784	-1.924	-1.256		3.823
S. minutus	-0.469	-0.120	0.555	-0.040	-0.020	0.920	

Supplementary Table S10 – Regression coefficients between pairs of species at the 95th percentile (Dark yellow) and 5th percentile (Light yellow) value of Precipitation of Warmest Quarter (Bio18). All other variables are at their average. The regression coefficients are symmetrical and provide the impact of the presence/absence of a species on the log-odds of the occurrence probability of the other species.

	S. etruscus	*C. leucodon*	*C. russula*	*C. suaveolens*	*S. araneus*	*S. coronatus*	*S. minutus*
S. etruscus		-3.046	4.253	2.578	0.991	3.791	0.733
C. leucodon	-0.594		-0.146	1.166	-0.561	-2.522	-0.152
C. russula	1.890	0.000		0.049	-1.219	2.054	-0.020
C. suaveolens	3.330	0.468	-0.436		-0.060	-3.728	-0.105
S. araneus	-2.706	-0.170	0.153	-1.679		8.390	-0.032
S. coronatus	-6.790	4.211	9.429	0.087	-4.583		2.116
S. minutus	-0.802	-0.066	0.011	-0.112	0.015	2.191	

Supplementary Table S11 – Regression coefficients between pairs of species at the 95th percentile (Dark yellow) and 5th percentile (Light yellow) value of Precipitation of Coldest Quarter (Bio19). All other variables are at their average. The regression coefficients are symmetrical and provide the impact of the presence/absence of a species on the log-odds of the occurrence probability of the other species.

	S. etruscus	*C. leucodon*	*C. russula*	*C. suaveolens*	*S. araneus*	*S. coronatus*	*S. minutus*
S. etruscus		-4.760	0.330	1.456	-3.026	-8.714	-0.921
C. leucodon	-0.454		0.066	-0.110	-0.303	4.974	-0.133
C. russula	4.413	-0.142		-0.203	-0.194	5.843	0.917
C. suaveolens	3.657	1.269	-0.182		-1.007	-0.152	-0.131
S. araneus	0.230	-0.401	-0.714	-0.780		-1.206	-0.060
S. coronatus	2.095	-1.222	5.585	-2.672	3.576		1.306
S. minutus	0.410	-0.099	-0.445	-0.098	0.015	2.557	

Supplementary Table S12 - Relative importance of variables in predicting the distribution of Suncus etruscus*. The species names in the variables refer to the presence of that species. x denotes interaction. The regression coefficient refers to the symmetrical regression coefficients that provide the impact of the presence/absence of a species on the log-odds of the occurrence probability of the other species. The variables are: Annual Mean Temperature (Bio 1), Mean Diurnal Range (Mean of the monthly maximum temperature subtracted by the monthly minimum temperature) (Bio 2), Mean Temperature of Wettest Quarter (Bio 8), Precipitation Seasonality (Bio 15), Precipitation of Warmest Quarter (Bio 18) and Precipitation of Coldest Quarter (Bio 19).*

Variable	Relative Importance	Regression Coefficient
Bio 15 x *S. coronatus*	0.121	5.008
Bio 8^2 x *C. suaveolens*	0.104	-4.643
Bio 1 x *S. coronatus*	0.092	4.358
Bio 8 x *C. suaveolens*	0.086	4.218
Bio 8 x *S. coronatus*	0.085	-4.179
Bio 2^2 x *S. coronatus*	0.081	-4.099
Bio 19 x *S. coronatus*	0.062	-3.578
Bio 1	0.044	3.031
C. russula	0.043	2.978
Bio 18 x *S. coronatus*	0.041	2.921
Bio 8^2 x *S. coronatus*	0.040	2.873
Bio 8 x *C. leucodon*	0.034	-2.634
Bio 8^2 x *C. leucodon*	0.022	2.108
C. suaveolens	0.018	1.946
Bio 1 x *S. minutus*	0.018	-1.917
Bio 1^2 x *S. minutus*	0.014	1.695
S. coronatus	0.013	-1.664

Supplementary Table S13 - Relative importance of variables in predicting the distribution of Crocidura leucodon. *The species names in the variables refer to the presence of that species. x denotes interaction. The regression coefficient refers to the symmetrical regression coefficients that provide the impact of the presence/absence of a species on the log-odds of the occurrence probability of the other species. The variables are: Annual Mean Temperature (Bio 1), Mean Diurnal Range (Mean of the monthly maximum temperature subtracted by the monthly minimum temperature) (Bio 2), Mean Temperature of Wettest Quarter (Bio 8), Precipitation of Warmest Quarter (Bio 18) and Precipitation of Coldest Quarter (Bio 19).*

Variable	Relative Importance	Mean Coefficient
Bio 2 x *S. coronatus*	0.350	-6.925
Bio 2^2 x *S. coronatus*	0.330	6.722
Bio 8 x *S. etruscus*	0.051	-2.634
Bio 1 x *S. coronatus*	0.035	2.190
Bio 8^2 x *S. etruscus*	0.032	2.108
Bio 19 x *S. coronatus*	0.031	2.051
Bio 1	0.027	1.937
Bio 1^2 x *S. coronatus*	0.025	-1.865
Bio 18 x *S. coronatus*	0.025	-1.859
Bio 19 x *S. etruscus*	0.015	-1.425
Bio 1 x *C. suaveolens*	0.013	-1.310

Supplementary Table S14 - Relative importance of variables in predicting the distribution of Crocidura russula. *The species names in the variables refer to the presence of that species. x denotes interaction. The regression coefficient refers to the symmetrical regression coefficients that provide the impact of the presence/absence of a species on the log-odds of the occurrence probability of the other species. The variables are: Annual Mean Temperature (Bio 1), Mean Diurnal Range (Mean of the monthly maximum temperature subtracted by the monthly minimum temperature) (Bio 2), Precipitation Seasonality (Bio 15), Precipitation of Warmest Quarter (Bio 18) and Precipitation of Coldest Quarter (Bio 19).*

Variable	Relative Importance	Mean Coefficient
S. coronatus	0.316	5.016
S. etruscus	0.111	2.978
Bio 1^2 x *S. coronatus*	0.085	-2.599
Bio 2 x *S. coronatus*	0.065	2.267
Bio 2^2 x *S. coronatus*	0.062	-2.219
Bio 15 x *S. coronatus*	0.053	2.046
Bio 18 x *S. coronatus*	0.052	-2.036
Bio 2 x *S. araneus*	0.046	-1.907
Bio 2^2 x *S. araneus*	0.033	1.618
Bio 1 x *S. coronatus*	0.031	1.578
Bio 19 x *S. etruscus*	0.023	-1.352
Bio 1^2 x *S. araneus*	0.022	-1.330
Bio 2^2	0.014	1.061
Bio 1 x *S. araneus*	0.013	1.024

Supplementary Table S15 - Relative importance of variables in predicting the distribution of Crocidura suaveolens. *The species names in the variables refer to the presence of that species. x denotes interaction. The regression coefficient refers to the symmetrical regression coefficients that provide the impact of the presence/absence of a species on the log-odds of the occurrence probability of the other species. The variables are: Annual Mean Temperature (Bio 1), Mean Diurnal Range (Mean of the monthly maximum temperature subtracted by the monthly minimum temperature) (Bio 2), Mean Temperature of Wettest Quarter (Bio 8) and Precipitation of Warmest Quarter (Bio 18).*

Variable	Relative Importance	Mean Coefficient
Bio 8^2 x *S. etruscus*	0.204	-4.643
Bio 8 x *S. etruscus*	0.168	4.218
Bio 2	0.150	3.979
Bio 1 x *S. coronatus*	0.136	-3.797
Bio 2^2	0.134	-3.771
S. etruscus	0.036	1.946
Bio 1	0.023	1.574
Bio 2 x *S. araneus*	0.021	-1.474
S. coronatus	0.017	-1.333
Bio 1 x *C. leucodon*	0.016	-1.310
Bio 2^2 x *S. araneus*	0.011	1.085
C. leucodon	0.011	1.073
Bio 1^2 x *S. etruscus*	0.011	-1.064
Bio 18 x *S. coronatus*	0.010	-1.053

Supplementary Table S16 - Relative importance of variables in predicting the distribution of Sorex araneus. *The species names in the variables refer to the presence of that species. x denotes interaction. The regression coefficient refers to the symmetrical regression coefficients that provide the impact of the presence/absence of a species on the log-odds of the occurrence probability of the other species. The variables are: Annual Mean Temperature (Bio 1), Mean Diurnal Range (Mean of the monthly maximum temperature subtracted by the monthly minimum temperature) (Bio 2), Mean Temperature of Wettest Quarter (Bio 8), Precipitation Seasonality (Bio 15), Precipitation of Warmest Quarter (Bio 18) and Precipitation of Coldest Quarter (Bio 19).*

Variable	Relative Importance	Mean Coefficient
Bio 1 x *S. coronatus*	0.141	-4.266
Bio 1^2 x *S. coronatus*	0.131	4.113
Bio 2 x *S. coronatus*	0.114	-3.844
Bio 2^2 x *S. coronatus*	0.112	3.803
Bio 18 x *S. coronatus*	0.099	3.582
Bio 1^2	0.069	-2.992
S. coronatus	0.061	2.817
Bio 15 x *S. coronatus*	0.042	2.331
Bio 2 x *C. russula*	0.028	-1.907
Bio 2^2 x *C. russula*	0.020	1.618
Bio 8	0.019	-1.586
Bio 19 x *S. coronatus*	0.019	-1.583
Bio 8^2	0.018	1.523
Bio 2 x *C. suaveolens*	0.017	-1.474
Bio 1^2 x *C. russula*	0.014	-1.330
Bio 2^2	0.010	-1.143

Supplementary Table S17 - Relative importance of variables in predicting the distribution of Sorex coronatus. *The species names in the variables refer to the presence of that species. x denotes interaction. The regression coefficient refers to the symmetrical regression coefficients that provide the impact of the presence/absence of a species on the log-odds of the occurrence probability of the other species. The variables are: Annual Mean Temperature (Bio 1), Mean Diurnal Range (Mean of the monthly maximum temperature subtracted by the monthly minimum temperature) (Bio 2), Mean Temperature of Wettest Quarter (Bio 8), Precipitation Seasonality (Bio 15), Precipitation of Warmest Quarter (Bio 18) and Precipitation of Coldest Quarter (Bio 19).*

Variable	Relative Importance	Mean Coefficient
Bio 15	0.106	-7.200
Bio 2 x *C. leucodon*	0.098	-6.925
Bio 2^2 x *C. leucodon*	0.092	6.722
C. russula	0.051	5.016
Bio 15 x *S. etruscus*	0.051	5.008
Bio 1 x *S. etruscus*	0.039	4.358
Bio 1 x *S. araneus*	0.037	-4.266
Bio 8 x *S. etruscus*	0.036	-4.179
Bio 1^2 x *S. araneus*	0.035	4.113
Bio 2^2 x *S. etruscus*	0.034	-4.099
Bio 2 x *S. araneus*	0.030	-3.844
Bio 2^2 x *S. araneus*	0.030	3.803
Bio 1 x *C. suaveolens*	0.029	-3.797
Bio 18 x *S. araneus*	0.026	3.582
Bio 19 x *S. etruscus*	0.026	-3.578
Bio 2^2	0.023	-3.369
Bio 18 x *S. etruscus*	0.017	2.921
Bio 8^2 x *S. etruscus*	0.017	2.873
S. araneus	0.016	2.817
Bio 2^2 x *S. minutus*	0.014	-2.642
Bio $1^{2\ x}$ *C. russula*	0.014	-2.599
S. minutus	0.011	2.364
Bio 15 x *S. araneus*	0.011	2.331
Bio 2 x *S. minutus*	0.011	2.283
Bio 2 x *C. russula*	0.011	2.267
Bio 2	0.010	2.221
Bio 2^2 x *C. russula*	0.010	-2.219

Supplementary Table S18 - Relative importance of variables in predicting the distribution of Sorex minutus. *The species names in the variables refer to the presence of that species. x denotes interaction. The regression coefficient refers to the symmetrical regression coefficients that provide the impact of the presence/absence of a species on the log-odds of the occurrence probability of the other species. The variables are: Annual Mean Temperature (Bio 1), Mean Diurnal Range (Mean of the monthly maximum temperature subtracted by the monthly minimum temperature) (Bio 2), Mean Temperature of Wettest Quarter (Bio 8) and Precipitation Seasonality (Bio 15).*

Variable	Relative Importance	Mean Coefficient
Bio 2^2 x *S. coronatus*	0.209	-2.642
S. coronatus	0.168	2.364
Bio 2 x *S. coronatus*	0.156	2.283
Bio 1 x *S. etruscus*	0.110	-1.917
Bio 1^2 x *S. etruscus*	0.086	1.695
Bio 8^2 x *S. coronatus*	0.045	1.229
Bio 1^2 x *S. coronatus*	0.042	1.189
Bio 8 x *S. coronatus*	0.026	-0.936
Bio 2^2	0.024	-0.894
Bio 15 x *S. coronatus*	0.023	0.874
Bio 8 x *S. etruscus*	0.020	-0.824
Bio 1^2	0.018	-0.772
Bio 1 x *S. coronatus*	0.014	-0.676
